# Early dynamics of the semantic priming shift

**DOI:** 10.2478/v10053-008-0126-9

**Published:** 2013-01-01

**Authors:** Frédéric Lavigne, Lucile Chanquoy, Laurent Dumercy, Françoise Vitu

**Affiliations:** 1 Laboratoire Bases, Corpus, Langage, UMR 7320, CNRS and Université de Nice-Sophia Antipolis, France; 2 Laboratoire de Psychologie Cognitive, UMR 7290, CNRS and Aix-Marseille Université, France

**Keywords:** association strength, concept, interstimulus interval, multiple priming, prime duration

## Abstract

Semantic processing of sequences of words requires the cognitive system to keep several word meanings simultaneously activated in working memory with limited capacity. The real- time updating of the sequence of word meanings relies on dynamic changes in the associates to the words that are activated. Protocols involving two sequential primes report a semantic priming shift from larger priming of associates to the first prime to larger priming of associates to the second prime, in a range of long SOAs (stimulus-onset asynchronies) between the second prime and the target. However, the possibility for an early semantic priming shift is still to be tested, and its dynamics as a function of association strength remain unknown. Three multiple priming experiments are proposed that cross-manipulate association strength between each of two successive primes and a target, for different values of short SOAs and prime durations. Results show an early priming shift ranging from priming of associates to the first prime only to priming of strong associates to the first prime and all of the associates to the second prime. We investigated the neural basis of the early priming shift by using a network model of spike frequency adaptive cortical neurons (e.g., Deco & Rolls, 2005), able to code different association strengths between the primes and the target. The cortical network model provides a description of the early dynamics of the priming shift in terms of pro-active and retro-active interferences within populations of excitatory neurons regulated by fast and unselective inhibitory feedback.

## Introduction

Language comprehension requires the cognitive system to activate in real time the meanings of several words. A consequence of the limited capacity of the working memory system (see Cowan, 2001; Haarmann & Usher, 2001) is that all the associates to the words read in a sequence cannot be activated, leading to only partial activation of the meaning of a given word. Selection processes are therefore involved that keep activated only the associates that best correspond to the sequence of words (Whitney, Grossman, & Kircher, 2009; Whitney, Jefferies, & Kircher, 2010). During the processing of a sequence of words, the activation of associates to the words would then shift - completely or partially - from associates to previously read words to associates to later read words. A late semantic shift has been put in evidence that obeys slow dynamics: Associates to a first word are initially activated quickly, but can be deactivated about 400 to 500 ms after the start of the subsequent (here: second) word (for a review, see Lavigne, Dumercy, & Darmon, 2011). Such dynamics are far slower than the natural reading speed of about five words per second would suggest: Fixation durations of about 200 ms per word during reading imply a faster pace. We therefore tested whether an earlier semantic shift could be demonstrated.

The activation of a target associate corresponds to shorter response times to a target word (e.g., *butter*) when a preceding prime word is related (e.g., *bread*) than when it is unrelated (e.g., *tree*; cf. Meyer & Schvaneveldt, 1971). In sequences of at least two primes, multiple priming processes are analyzed by recording response times to a target (e.g., *tiger*) that is (a) related to the two preceding primes (RR condition; e.g., *lion* and *stripes*), (b) unrelated to the primes (UU condition; e.g., *fuel* and *shutter*), (c) related to the first prime (RU condition; e.g., *lion and shutter*), or (d) related to the second prime (UR condition; e.g., *fuel* and *stripes*). Multiple priming effects are calculated by subtracting reaction times in a given condition of primes-target relatedness (RR, RU, UR) to reaction times in the UU baseline condition (McNamara, 1992). Thus, RR, RU, or UR priming effects are measured as the difference between response times in the UU condition and response times in the RR, RU, or UR condition, respectively. Comparison of priming effects in these three conditions provides a measure of the dynamics of the meanings activated by a given sequence of words as they unfold over time in working memory (e.g., Balota & Paul, 1996; Masson, 1995; Masson, Besner, & Humphreys, 1991; McNamara, 1992; Whitney et al., 2009, 2010). However, the respective levels of priming in these three conditions can greatly vary over time (Lavigne et al., 2011; Lavigne & Vitu, 1997). These variable dynamics owe to the level of activation of the target as a function of its relatedness to the primes, but also to the intervals between processing of the words in the sequence.

### Behavioral correlates of activation and interference

The timing of multiple-priming protocols is defined by two SOAs (stimulus-onset asynchronies): SOA1 is the delay between Prime 1 and Prime 2 onsets, and SOA2 is the delay between Prime 2 and target onsets. A recent meta-analysis of multiple-priming effects showed that while UR priming of associates to the second prime is stable over a large range of SOAs, RU priming of associates to the first prime disappears when SOA2 becomes longer than SOA1 (Lavigne et al., 2011). Results indicate that for short SOA1 and long SOA2, UR priming becomes larger than RU priming. In addition, there is a semantic priming shift, as measured by the difference between UR and RU priming, from activation of RU associates to the first prime to activation of UR associates to the second prime. The priming shift, defined as the *UR-RU priming difference*, could rely on dynamic changes of the balance between activation of associates to a related prime, and selection among the set of associates that are activated.

Sequences of items such as those displayed in priming protocols are processed in a limited capacity working memory system (e.g., Cowan, 2001). As a consequence, when maximum capacity is reached, interference perturbs the processing of the items (Amit, Bernacchia, & Yakovlev, 2003; see Haarmann & Usher, 2001, for a review). The processing of items generates proactive interference that can perturb the processing of subsequent items and, to a larger extent, retroactive interference that can perturb the processing of preceding items. Interference is reported in the experimental literature to affect semantic priming (Deacon, Uhm, Ritter, Hewitt, & Dynowska, 1999; Hutchison, Neely, & Johnson, 2001; Neely, 1976, 1977, 1991; Neely, Keefe, & Ross, 1989; Rastle, Davis, Marslen-Wilson, & Tyler, 2000). In multiple-priming protocols, the limited capacity of the workingmemory system implies a selection of which RU and/or UR associates are activated and which are not (Kandhadai & Federmeier, 2007; Lavigne et al., 2011; Lavigne & Vitu, 1997; Whitney, Grossman, & Kircher, 2009). Selection among RU and UR associates to the first and second primes, respectively, can be achieved by the combined effects of their activation through semantic associations with related prime(s), and selection that arises as a result of the interference generated by the unrelated prime.

With a single prime, the amount of activation received by a target is reported in the literature as increasing with the strength of its association to a related prime (Abernethy & Coney, 1993; Coney, 2002; Frishkoff, 2007; Hutchinson, Whitman, Abeare, & Raiter, 2003) and with the prime-target SOA (Coney, 2002; Rastle et al., 2000; for reviews, see Brunel & Lavigne, 2009; Chiarello, Liu, Shears, Quan, & Kacinik, 2003). A recent experimental study has revealed that the slow dynamics of the priming shift in the long SOA range depend on the strength of the association between the target and its related prime (Lavigne, Dumercy, Chanquoy, Mercier, & Vitu, 2012). An RU target strongly associated to its related Prime 1 becomes rapidly activated and stays activated longer after processing of the unrelated Prime 2. However, with a long SOA2 of 650 ms, an RU associate to the first prime becomes less activated than a UR associate to the second prime, corresponding to a slow priming shift for strong associates. This time course is in accordance with the literature on slow deactivation of a previously activated target. However, Lavigne et al.’s (2012) study also reports that RU targets weakly associated to Prime 1 are not activated if an unrelated Prime 2 is processed before the target during a short SOA2 (250 ms), while weakly associated targets are activated at short SOAs by a related prime in absence of any Prime 2 (e.g., Coney, 2002). This suggests that, in multiple priming, the unrelated Prime 2 could trigger fast selection processes at short SOAs, and that an early priming shift could deactivate at least weak RU associates. However, the lack of experimental data in the short SOA range limits the possibility to assess for fast selection processes and to identify the early determinants of the semantic priming shift.

### Neural correlates of activation and interference

The processes of activation and interference are also revealed by neural activities recorded in behaving monkeys during the processing of associated prime and target images (e.g., Miyashita, 1988; Miyashita & Chang, 1988; Rainer et al., 1999; Sakai & Miyashita, 1991). Electrophysiological studies report that spike rates of neurons coding for a stimulus exhibit four main types of activities depending on the events considered during the protocol: (a) spontaneous low-frequency activity in the absence of the stimulus (e.g., Lehky, Kiani, Esteky, & Tanaka, 2011); (b) a high-frequency perceptual response during and immediately following the stimulus (e.g., Lehky et al., 2011; Miller & Desimone, 1994); (c) retrospective activity at an intermediate firing rate after the stimulus (Fuster, 2001; Miller & Desimone, 1994; Naya, Yoshida, & Miyashita, 2001, 2003; Rainer, Rao, & Miller, 1999), supposed to underlie activity of the stimulus in working memory (Amit & Brunel, 1997); and (d) prospective activity at an intermediate firing rate in the absence of the stimulus but when an associated stimulus is presented. Prospective activity starts at spontaneous activity level and increases during the prime-target SOA (Miyashita, 1988; Miyashita & Chang, 1988; Rainer et al., 1999; Sakai & Miyashita, 1991) to generate priming effects (Erickson & Desimone, 1999).

These different types of neuronal activities are reproduced by computational models based on realistic biophysical properties of the cortical neurons and architectural properties of the cerebral cortex (see e.g., Amit, 1995; Amit & Brunel, 1997). Such cortical network models account for spontaneous, perceptual, and retrospective activities at the level of populations of inter-connected neurons (Amit et al., 2003; Brunel & Wang, 2001; Renart, Moreno, de la Rocha, Parga, & Rolls, 2001), and for prospective activity of neurons coding for the target, that increases with SOA through selective activation by associated neurons coding for the prime (Brunel, 1996; Lavigne, 2004; Lavigne & Darmon, 2008; Lavigne & Denis, 2001, 2002; Mongillo, Amit, & Brunel, 2003). These models also show that the combination of these activities generates a large variety of single word priming effects as reported in humans, as a function of the SOA, various types of semantic relations, and different values of association strength (Brunel & Lavigne, 2009).

In cortical network models, excitatory neurons coding for items also activate inhibitory interneurons that provide regulatory negative retro-action preventing runaway excitation of the entire network (e.g., Amit, Bernacchia, & Yakovlev, 2003; Amit & Brunel, 1997). Therefore, in multiple priming, processing of the sequential primes and their activated associates generates unselective inhibition proportional to the overall level of activation. Such inhibitory feedback limits the number of simultaneously activated items (Haarmann & Usher, 2001), and thereby constrains the capacity of the network’s working memory (Cowan, 2001). As a consequence, the activity of a target increases with the SOA following a related prime, but also decreases with the duration of the strong inhibitory feedback by an unrelated prime (Lavigne et al., 2011). In this model, the inhibition of a population of neurons (e.g., coding for an RU target) accounts for the slow priming shift at long SOAs, which is mainly due to slow spike frequency adaptation. However, neurophysiological data report that inhibitory post-synaptic potentials triggered by gabaergic neurons obey fast dynamics of the order of milliseconds (Salin & Prince, 1996; Xiang, Huguenard, & Prince, 1998). Such a fast inhibitory feedback would cause rapid interference in working memory (Brunel & Wang, 2001), regulating priming effects even at short SOAs (see Brunel & Lavigne, 2009, for a discussion). The level of inhibition is proportional to the level of activation of the prime, which itself depends on the duration of its processing. The prime is more strongly activated during its processing (“perceptual response”) than during the ISI (interstimulus interval) without stimulus (Mongillo et al., 2003). These mechanisms suggest that the priming shift could also occur at short SOAs and is not limited to the long SOA range. Such an early priming shift would be based on fast inhibitory feedback elicited by the prime, but under the condition that SOA2 is longer than SOA1; indeed, this would allow facilitation by the related Prime 2 in the UR priming condition to win out against the proactive interferenceelicited by the unrelated Prime 1.

In conclusion, the cortical network model allows us to describe the results of the experiments in terms of neural mechanisms of priming and prime-elicited interference (see the Model Behavior section).

### Early semantic priming shift

The current study investigated the early semantic priming shift in the short SOA range where multiple-priming effects are poorly understood. An early shift at short SOAs would allow for proactive interference by the unrelated Prime 1 to prevent priming by the related Prime 2 in the UR priming condition where SOA2 is shorter than SOA1 (Experiment 1). It would also allow for an increase of net priming by the related Prime 2 in the UR priming condition when SOA2 becomes longer than SOA1 (Experiment 2). Finally, early semantic shifts entail the possibility for UR priming to activate associates to the Prime 2 that deactivate associates to the Prime 1 if a longer Prime 2 duration generates stronger retroactive inhibition. As a consequence, UR priming could potentially completely cancel RU priming, which is tested in Experiment 3. The behavioral results of these experiments are described within the framework of the cortical network model, in terms of processes of reciprocal activation and inhibition between (populations of) neurons coding for the primes and target, respectively.

## Methods of the experiments

### Participants

A total of 271 participants (189 females and 82 males between 18 and 45 years old) participated in the three experiments in exchange for course credit at the University of Nice-Sophia Antipolis. All participants were French native speakers and had normal or corrected-to-normal visual acuity. Each participant took part in only one experiment.

### Apparatus

Experiments were run on a computer using E-prime software to control stimulus presentation and to record participants’ response times and errors. Participants were seated at 60 cm from a 15-inch PC monitor where stimuli were displayed.

### Material

The stimulus material consisted of 96 word triplets (two word primes and one word target) and 96 pseudo-word triplets (two word primes and one pseudo-word target). All triplets were constructed on the basis of the relation between the two primes and the target words (from free production norms in French; see e.g., Cornuéjols, 1999; Ferrand & Alario, 1998; Kurzepa, 2003). All 96 word triplets were selected so that the target was related to each of the two primes, while the two primes were unrelated. These 96 triplets corresponded to four groups of 24 triplets in which each prime could be either weakly (w) or strongly (s) related to the target. The association strength was calculated as being the percentage of production of a target word in relation to a given prime word among all participants. The average strength was 33.1% (between 15.7 and 92.1%) for strong associates and 8.6% (between 3.3 and 14.6%) for weak associates. The choice for these ranges of strengths allowed us to keep as many triplets as possible for the experiments. The combination of two association strengths and two primes gene-rated four conditions of association strength within a triplet: (a) both primes strongly related to the target (ss; e.g., *guitar* and *harp* for *string*); (b) the first prime strongly related and the second prime weakly related to the target (sw; e.g., *bonnet* and *pullover* for *wool*); (c) the first prime weakly related and the second prime strongly related to the target (ws; e.g., *cradle* and *doll* for *child*); and (d) both primes weakly related to the target (ww; e.g., *cherry* and *apricot* for *pit*).

Targets involved in the four conditions of association strength were controlled in lexical frequency (Lexique 3.5; New, Pallier, Brysbaert, & Ferrand, 2004); this did not differ between the ss, ws, sw, and ww conditions (mean frequencies of 90, 60, 145, and 95 occurrences per million, respectively; pairwise *t*-tests *p*> .05). All primes and targets words were between three and nine characters in length, and the lengths and frequencies of prime words did not significantly differ between the four conditions of association strength. For a given value of association strength, the same target word was tested in the RR, RU, UR, and UU conditions. This allowed us to test priming effects (UU compared to RR, RU, or UR) on the same targets for a given strength. However, it was not possible to cross manipulate four related primes in the four conditions of strength on the same target (i.e., two strongly and two weakly related primes). Targets in the ss, sw, ws, and ww conditions were therefore different words. To prevent the lexical properties of the target word themselves to possibly have slightly different effects between conditions of strength, the UU conditions were not aggregated across conditions of strength.

In each of the 96 word triplets selected, the two primes were related to the target. By replacing the first, second, both, or none of the primes in a triplet by an unrelated word (chosen among the set of primes related to other targets), each target could be embedded in four different combinations of relatedness between prime and target, thus defining four conditions of relatedness: (a) both primes related to the target (RR; e.g., *hedgehog* and *cactus* for *spine*); (b) the first prime related, and the second prime unrelated to the target (RU; e.g., *hedgehog* and *cherry* for *spine*); (c) the first prime unrelated, and the second prime related to the target (UR; e.g., *doll* and *cactus* for *spine*); (d) both primes unrelated to the target (UU; e.g., *cherry* and *doll* for *spine*). The Primes 1 and 2 were related indirectly through their common associated target in the RR condition, but the absence of a direct relation between them was controlled based on the production norms.

The two independent variables of within-items primes-target relatedness (RR, RU, UR, UU) and of between-items association strength (ss, sw, ws, ww) were cross-manipulated in order to obtain 16 experimental conditions. Eight experimental lists of 96 word triplets and 96 pseudo-word triplets were created to counterbalance primes and targets across conditions of relatedness, so that each word appeared only once on the list of triplets presented to a participant. In half of the triplets on a list (96), the target was a bona fide French word (e.g., “épine” = *spine*). In the other half of the triplets (96) the target was a pseudo-word derived from a word by replacing one or two letters, but constructed in accordance with the phonotactic constraints of French (e.g., “soudis” derived from *souris* = *mouse*).

### Design, task, and procedure

A 4 × 4 factorial design was used, with Relatedness (RR, RU, UR, UU) being manipulated as a within-participants and within-items variable, and with Associative Strength (ss, sw, ws, ww) being manipulated as a within-participants and between-items variable.

Participants were tested individually in a soundproof room. They were told that on each trial, three stimuli would be presented in a sequence at the center of the screen: two words in lowercase lettering followed by a word or a pseudo-word in uppercase lettering. They were asked to perform a lexical decision task by responding as quickly and as accurately as possible with their dominant hand to indicate whether the third letter string was a French word (left click on the computer mouse) or not (right click). Sixteen practice trials were presented, followed by two experimental blocks of 96 trials, allowing a break between blocks. The order in which trials were presented was randomized and changed for each participant.

### Protocol

Stimuli were displayed in 18-point Courier New black font centered on a white background, with primes in lowercase lettering and targets in uppercase lettering. Each trial consisted of the following sequence of events: a blank screen for 800 ms, a visual warning signal made of three horizontally displayed asterisks for 200 ms, a blank screen for 400 ms; SOA1 corresponding to the presentation of the first prime for a duration PD1, followed by the first interstimuli interval (ISI1; a blank screen); SOA2 corresponding to the presentation of the second prime for a duration PD2, followed by the second interval (ISI2; a blank screen); finally, the target was presented for 240 ms, followed by a blank screen until the participant’s response. Response times and errors were recorded for each participant’s response.

The corresponding SOAs and ISIs of the experiments were specified as follows:

Experiment 1: PD1 = 50 ms, ISI1 = 200 ms (SOA1 = 250 ms), PD2 = 50 ms, ISI2 = 75 ms (SOA2 = 125 ms).

Experiment 2: PD1 = 50 ms, ISI1 = 75 ms (SOA1 = 125 ms), PD2 = 50 ms, ISI2 = 200 ms (SOA2 = 250 ms).

Experiment 3: PD1 = 150 ms, ISI1 = 100 ms (SOA1 = 250 ms), PD2 = 150 ms, ISI2 = 100 ms (SOA2 = 250 ms).

### Data analyses

**Figure 1.**
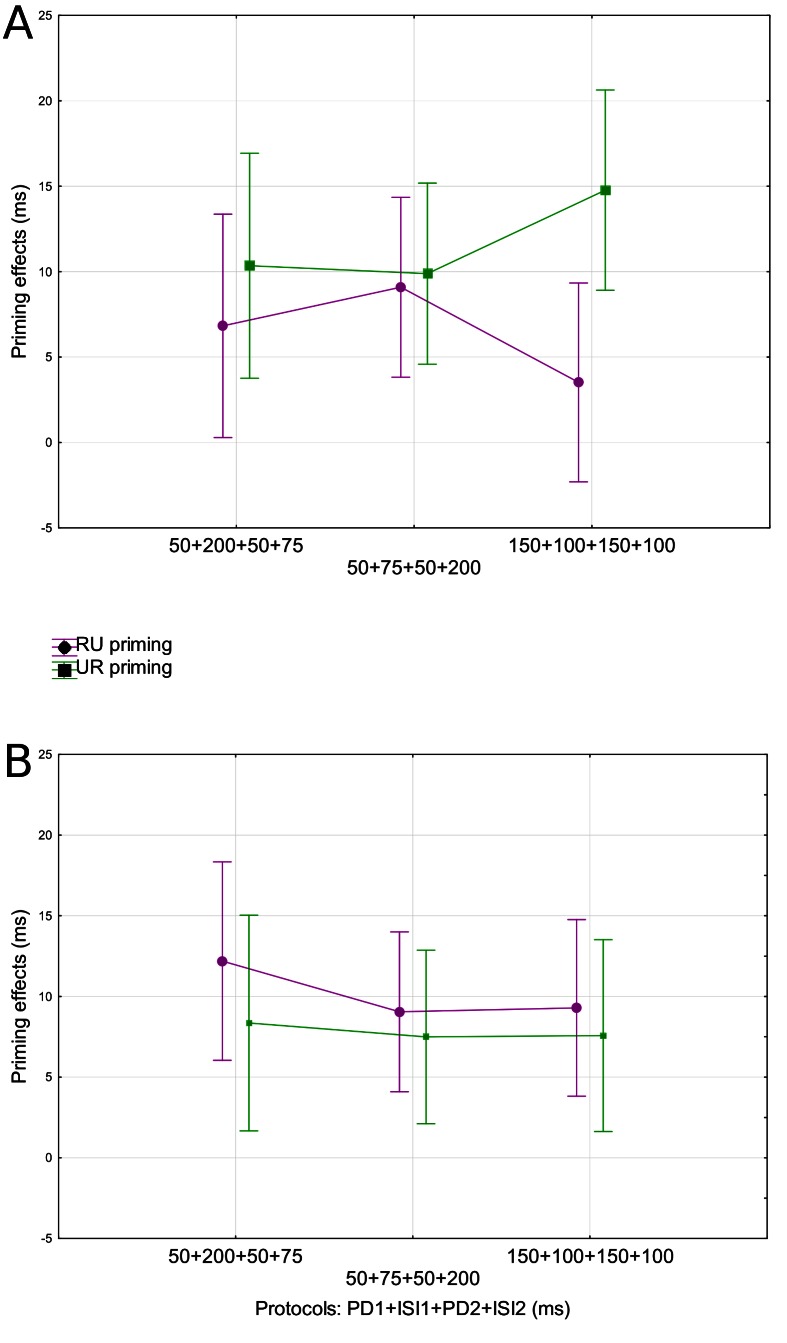
Semantic priming effects in the RU (a condition in which a target is related to the first prime; violet circles) and UR (a condition in which a target is related to the second prime; green squares) conditions as a function of the three experimental protocols and for weak associates (Panel A) and strong associates (Panel B). Mean values and standard errors are displayed. ISI = inter-stimuli interval.

The main data analyses of the RR, RU, and UR priming effects relative to the UU baseline were conducted, using a 4 × 4 design with four conditions of Relatedness and four conditions of Association Strength. Analyses of variance were performed on Response Times (RTs) associated to correct responses on words, with Relatedness (four conditions: RR vs. RU vs. UR vs. UU) as a within-participants and within-items variable, and Association Strength (four conditions: ss vs. sw vs. ws vs. ww) as a within-participants and between-items variable. Trials in which participants made errors were excluded from the analyses. A cut-off was set for each participant at ±2.5 standard deviation units from each participant’s mean RT on words, and outliers were excluded from the analyses. Data from participants who made more than 15% of errors were also excluded from the analyses. Planned comparisons were performed on RU and UR priming. Given that in the RU and UR conditions the strength between the target and one related prime was considered (the first in RU priming and the second in UR priming), contrast analyses permitted to aggregate RU-ss and RU-sw to test for RU-s priming, RU-ws and RU-ww to test for RU-w priming, UR-ss and RU-ws to test for UR-s priming, and UR-sw and RU-ww to test for UR-w priming, with the same aggregations in the UU conditions. For the planned comparisons, this defined a 2 × 2 design: 2 (RU vs. UU) × 2 (ss & sw vs. ws & ww) for RU priming, and 2 (UR vs. UU) × 2 (ss & ws vs. sw & ww) for UR priming. This allowed us to test RU and UR priming by using 12 items per condition, by testing response times in the RU and UR conditions against response times on the same target items in the UU condition (see Figure 1). All p-values of significant effects for participants analyses (F1) and items analyses (*F_2_*, provided in the Appendix A) are indicated as lesser than .01 or than .001, and exact p-values are provided when greater than .01.

## Methods of the Cortical Network model

We propose here to link the types of priming shift investigated in the experiments to the underlying neuronal mechanisms of activation and inhibitory interference at work in a cortical network model. In Lavigne et al.’s (2011) spike frequency adaptive model of multiple priming, Association Strengths 1 and 2 were not cross-manipulated and prime durations were not varied, leaving open the question of their effects on the priming shift. We therefore generalized the model by cross-manipulating Association Strengths 1 and 2 between Prime 1 and the target, and between Prime 2 and the target, respectively - as it was the case in the current experiments - and by simulating the protocols of the experiments with the same short prime durations and ISIs.

### Network architecture and neurons dynamics

Here, we used a mean-field approach describing the firing of populations of neurons whose dynamics are described by average firing rates obeying a standard Wilson-Cowan type equation (Brunel & Lavigne, 2009; Brunel & Wang, 2001; see also Amit & Brunel, 1997). The network embeds 99 populations of excitatory neurons coding for the items, including the primes and target. These populations have a non-linear transfer function giving the firing rate versus the mean input current calculated as the sum of selective recurrent activities from the population and other populations in the network, selective external stimuli, spike frequency adaptation, and non-selective feedback inhibition. In the network, both a non-selective background state and selective attractors are present that correspond to a single activated item or to multiple activated items. The network includes multiple associated sets of neural populations whose synapses are potentiated to reproduce the same experimental conditions of primes-target relatedness (RR, RU, UR, and UU) for strong and weak primes-target associations (ss, sw, ws, ww). Numerical simulations used the same prime durations and ISIs as those tested in the experimental protocols.

### Data analyses

**Figure 2.**
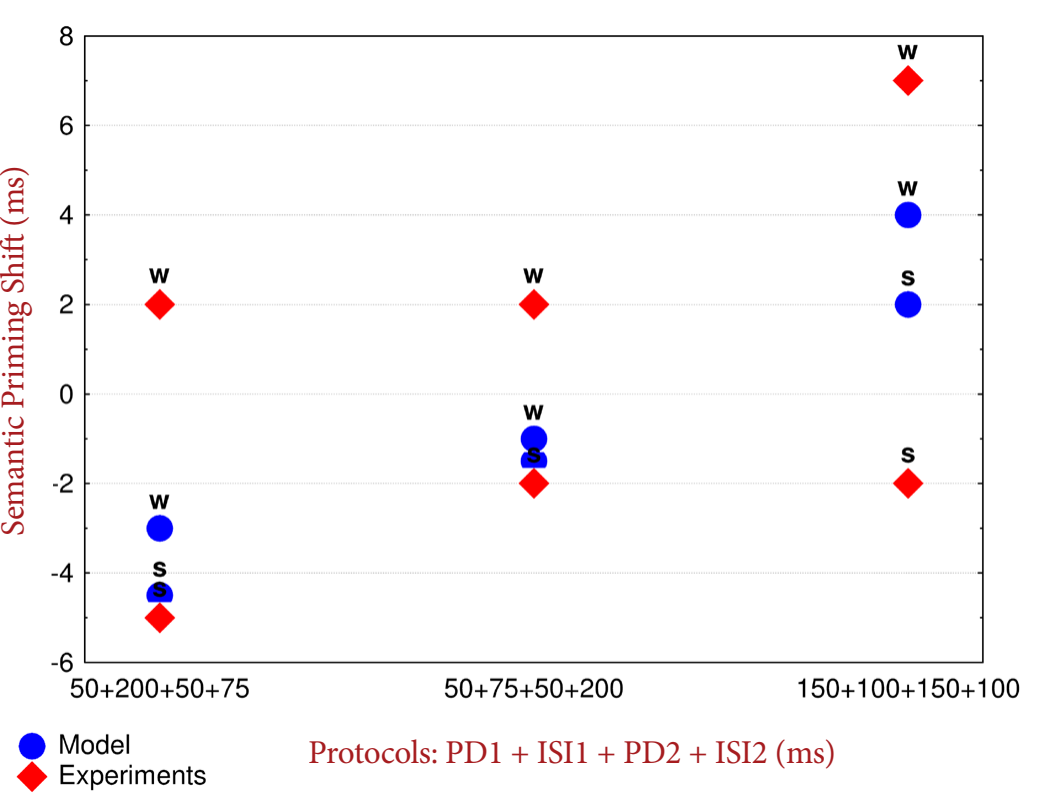
Semantic priming shift (UR-RU effects) as a function of the three experimental protocols. Mean values of the priming shift (for strong and weak associates) are displayed for the experiments (red rhombus) and for the model (blue circles). ISI = inter-stimuli interval. PD = Prime Duration. RU = a condition in which a target is related to the first prime. UR = a condition in which a target is related to the second prime.

The reaction time *T*^Ɵ^ for each condition was the time that elapsed from target onset to the instant at which the mean firing rate of the corresponding neuron population crossed a threshold ν^Ɵ^ for the first time. This was based on electrophysiological studies in monkeys reporting a correlation between spike rates and response times (Roitman & Shadlen, 2002), as well as shorter reaction times on targets preceded by an associated prime (Erickson & Desimone, 1999). In the simulations, the RR, RU, and UR experimental conditions gave specific recognition times *T*^Ɵ^; each was subtracted to the recognition time in the UU condition in order to calculate the respective priming effects of each experiment. *T*^Ɵ^ depended on the level of activity of the population of neurons coding for the target at target onset, itself depending on its sources of activation from the related prime(s) and inhibition by non-selective inhibitory feedback. As a consequence, the magnitude of the multiple-priming effects depended on the conditions of relatedness and association strength embedded in the synaptic matrix, and on the specific protocol (see Table 1). This led to modeled magnitudes of the priming shift (UR-RU priming effects) that varied in a qualitatively similar way to those reported in the experiment: larger shifts for weak than for strong associates and increasing shifts from Protocol 1 to Protocol 3 (see Figure 2). The values of association strength used in the model to fit the current experiments were not specified ad hoc. Rather, we present simulation data using exactly the same values of association strength as in Lavigne et al.’s (2012) model that fitted experimental data on the slow priming shift at long SOAs. It is then possible to generalize the model’s behavior to a wide range of SOAs from 125 ms (current Experiments 1 and 2) to 650 ms (Lavigne et al., 2012).

**Table 1.** Mean RU and UR Priming Effects (Milliseconds) in the Model’s Simulations of the Three Protocols for Strong and Weak Associates

Protocol		50 + 200 + 50 + 75		50 + 75 + 50 + 200		150 + 100 + 150 + 100	
Strength		s	w	s	w	s	w
Relatedness	RU	14	11	14	12	14	9
	UR	9	8	12	11	16	13

*Note*. RU = a condition in which a target is related to the first prime. UR = a condition in which a target is related to the second prime. s = strong association strength. w = weak association strength.

## Experiment 1: 50 + 200 + 50 + 75

The respective amounts of activation of the RU and UR associates that subtend the priming shift result in the balance between activation received from their associated prime (first and second prime, respectively) and interference generated by the processing of the unrelated prime (second and first prime, respectively). The amounts of activation and interference themselves depend on the SOAs during which each prime can activate its associated target, and generate proactive or retroactive interference (the latter two on the basis of the priming of different words than the actual target). The purpose of Experiment 1 was to investigate multiple-priming effects in the range of short SOAs not yet cross-manipulated in multiple-priming experiments. Though UR priming is reported to be as reliable for a wide range of SOAs, experimental data are still lacking at very short SOA2s of less than 150 ms. At these short SOA2s, cortical network modeling predicts that net UR priming does not arise, due to proactive interference and too short an SOA2 for the Prime 2 to activate its UR associate. To test for the possibility that UR priming takes time to arise against proactive interference, SOA1 was set at 250 ms, and was thus longer than SOA2, set at 125 ms.

### Participants and protocol

Experiment 1 involved 60 participants and the following protocol:

PD1 = 50 ms, ISI1 = 200 ms (SOA1 = 250 ms), PD2 = 50 ms and ISI2 = 75 ms (SOA2 = 125 ms).

### Results

Data from three participants who made more than 15% errors were excluded from the analysis. Of the remaining 57 participants, the average error rate was 4.5% and data from error trials were excluded from the analysis. A two-way ANOVA was performed on RTs associated to correct responses on words, between the four conditions of Relatedness and four conditions of Association Strength (see Table 2).

**Table 2.** Mean Response Times (in Milliseconds) in Experiment 1 (50 + 200 + 50 + 75) as a Function of Primes-Target Relatedness (RR, RU, UR, UU) and Association Strength (ss, sw, ws, ww)

Association strength		ss	sw	ws	ww
Relatedness	RR	609 (91)	620 (94)	663 (93)	619 (73)
	RU	625 (91)	618 (91)	633 (92)	621 (86)
	UR	613 (83)	619 (83)	636 (87)	633 (86)
	UU	631 (86)	637 (88)	634 (89)	633 (80)

*Note*. Standard deviations in parentheses. RR = a condition in which a target is related to the two preceding primes. RU = a condition in which a target is related to the first prime. UR = a condition in which a target is related to the second prime. UU = a condition in which a target is unrelated to the primes. s = strong association strength. w = weak association strength.

Results show a main effect of relatedness, *F_1_*(3, 168) = 4.94, *MSE* = 2031, *p *< .01; a marginally significant effect of association strength, *F_1_*(3, 168) = 2.32, *MSE* = 2657, *p* = .077; and no significant interaction, *F_1_*(9, 504) = 0.91, *MSE* = 2472, *p* = .52.

Planned comparisons indicate significant RR priming effects, *F_1_*(1, 56) = 17.03, *MSE* = 1743.03, *p *< .001, showing that during the successive SOA1 and SOA2, the primes, or one of them have time to activate their (jointly) associated target.

Regarding the semantic priming shift, the overall RU priming effects are significant, *F_1_*(1, 56) = 5.47, *MSE* = 1885.59, *p* = .023. However, priming of strong RU associates is only marginally significant and priming of weak RU associates is not significant, *F_1_*(1, 56) = 3.37, *MSE* = 2512.09, *p* = .072, and *F_1_*(1, 56) = 1.34, *MSE* = 1982.72, *p* = .25, respectively. This indicates that the total SOA of 375 ms between Prime 1 and target onset (SOA1 + SOA2) is long enough to allow for overall RU priming despite retroactive interference by the unrelated Prime 2. This is in accordance with Lavigne et al.’s (2011) meta-analysis, at least for strong RU associates, and in agreement with the model’s simulations.

Regarding the UR condition, the overall priming effect is only marginally significant, *F_1_*(1, 56) = 3.6, *MSE* = 2728.41, *p* = .07, UR priming being significant for strong associates, *F_1_*(1, 56) = 4.62, *MSE* = 2046.36, *p* = .036, but not for weak associates, *F_1_*(1, 56) = 2.15, *MSE* = 2825.04, *p* = .15. For this range of short SOAs (so far not tested in the literature), the manipulation of association strength in the current study showed that UR priming depends on association strength. Experiment 1 suggests for the first time that UR priming, though being very reliable in the literature, is not ubiquitous at all SOAs for weak associates. When no other word is interposed between the related prime and target (as is the case in UR priming), single priming from one strongly associated prime arises at very short SOAs of a few tens of milliseconds (Perea & Gotor, 1997; Perea & Rosa, 2002; Rastle et al., 2000). The non-significant UR priming of weak associates in the current Experiment is then a new result, to be related to the slower activation of weak compared to strong associates in single priming (Coney, 2002) and/or to the proactive interference generated by the first prime unrelated to the UR target in multiple priming (Lavigne et al., 2011). The possibility for an increase of UR priming at a longer SOA2, and for a resulting interaction between RU and UR priming, will be tested in Experiment 2 and by comparing Experiments 1 and 2.

### Model behavior

In the network model, a trial begins with populations of spontaneous low activity. At Prime 1 onset, the activity of the corresponding neuron population increases (“perceptive response”). The level of activation decreases after the Prime 1 offset but remains active above the level of spontaneous activity based on retrospective activity in working memory. The first prime can therefore activate its associated target in the RU condition and generates priming effects compared to an unrelated prime in the UU condition (12-ms RU priming; cf. Table 4). In addition, the non-selective inhibitory feedback by Prime 1 limits the activation of further items due to proactive interference (Amit et al., 2003; Cowan, 2001; see Haarmann & Usher, 2001, for a review). This limiting inhibition by any Prime1 implies that Prime 2 activation is subject to proactive interference by Prime 1. This in turn prevents much of the priming effect of Prime 2 in the UR condition during the very short SOA2, leading to priming of low magnitude of UR condition’s Prime 2 unless a longer SOA2 was used (8 ms; see Table 4). In accordance with the results of Experiment 1, the model indicates that no priming shift has occurred yet when SOA1 is short but longer than SOA2, with an activation of the meaning of Prime 1 and a weaker activation of the meaning of Prime 2.

**Table 4.** Mean Response Time (in Milliseconds) in Experiment 3 (150 + 100 + 150 + 100) as a Function of Primes-Target Relatedness (RR, RU, UR, UU) and Association Strength (ss, sw, ws, ww)

Association strength		ss	sw	ws	ww
Relatedness	RR	579 (90)	582 (82)	585 (91)	581 (90)
	RU	580 (94)	589 (81)	595 (92)	601 (96)
	UR	573 (94)	589 (89)	593 (97)	591 (83)
	UU	586 (75)	602 (89)	595 (97)	608 (89)

*Note*. Standard deviations in parentheses. RR = a condition in which a target is related to the two preceding primes. RU = a condition in which a target is related to the first prime. UR = a condition in which a target is related to the second prime. UU = a condition in which a target is unrelated to the primes. s = strong association strength. w = weak association strength.

## Experiment 2: 50 + 75 + 50 + 200

Given that priming effects increase with SOA, Experiment 2 was designed to confirm that the condition with SOA2 longer than SOA1 allows for stronger UR priming, generating in turn stronger retroactive interference on the RU associates and an early priming shift. To test this possibility, the values of SOA1 and SOA2 were reversed in Experiment 2 compared to Experiment 1, with the SOA1 set at 125 ms and the SOA2 set at 250 ms, while the same primes durations as in Experiment 1 (50 ms) were used.

### Participants and protocol

Experiment 2 involved 89 participants and the following protocol:

PD1 = 50 ms, ISI1 = 75 ms (SOA1 = 125 ms), PD2 = 50 ms and ISI2 = 200 ms (SOA2 = 250 ms).

### Results

Data from one participant who made more than 15% errors were excluded from the analysis. Of the remaining 88 participants, the average error rate was 5.2%, and data from error trials were excluded from the analysis. A two-way ANOVA was performed on RTs between the four conditions of Relatedness and the four conditions of Association Strength (see Table 3).

**Table 3.** Mean Response Time (in Milliseconds) in Experiment 2 (50 + 75 + 50 + 200) as a Function of Primes-Target Relatedness (RR, RU, UR, UU) and Association Strength (ss, sw, ws, ww)

Association strength		ss	sw	ws	ww
Relatedness	RR	563 (92)	574 (102)	568 (83)	572 (93)
	RU	565 (88)	578 (96)	573 (94)	581 (82)
	UR	567 (93)	583 (87)	575 (92)	571 (85)
	UU	573 (97)	588 (97)	584 (93)	588 (94)

*Note*. Standard deviations in parentheses. RR = a condition in which a target is related to the two preceding primes. RU = a condition in which a target is related to the first prime. UR = a condition in which a target is related to the second prime. UU = a condition in which a target is unrelated to the primes. s = strong association strength. w = weak association strength.

Results show a main effect of relatedness, *F_1_*(3, 261) = 4.83, *MSE* = 2403, *p *< .01; a significant effect of association strength for participants, *F_1_*(3, 261) = 6.08, *MSE* = 2071, *p *< .001; and no significant interaction, *F_1_*(9, 783) = 0.41, *MSE* = 2472, *p* = .93.

Planned comparisons indicate significant RR priming effects, *F_1_*(1, 87) = 11.48, *MSE* = 2580.07, *p *< .01, showing that, as in Experiment 1, during the successive SOA1 and SOA2 the primes have time to activate their jointly associated target.

Regarding the priming shift, overall RU priming effects are significant, *F_1_*(1, 87) = 6.14, *MSE* = 2355.59, *p* = .015. The priming of both strong and weak RU associates was marginally significant, *F_1_*(1, 87) = 2.99,*MSE* = 2410.44, *p* = .087, and *F_1_*(1, 87) = 3.31, *MSE* = 2195.50, *p* = .072, respectively. This indicates that RU associates can be activated during the short 125-ms SOA1, in accordance with the literature reporting early single priming effects. With the short 50-ms Prime 2 duration, weak RU associates continue to activate targets against retroactive interference during the 200-ms ISI2. The overall UR priming effects are significant, *F_1_*(1, 87) = 5.38, *MSE* = 2469.44, *p* = .023. Here compared to Experiment 1, the priming of weak UR associates becomes significant, *F_1_*(1, 56) = 3.99, *MSE* = 2150.09, *p* = .049, subtending a priming shift between weak associates, in accordance with the marginally significant interaction between experiments (Experiment 1 vs. 2) and type of associates (RU vs. UR) for weak associates, *F_1_*(1, 143) = 3.57, *MSE* = 2422.89, *p* = .060 (the interaction is not significant for strong associates). This suggests that in Experiment 2, the 125-ms SOA1 was too short to allow for proactive interference, and the 250-ms SOA2 was long enough for UR priming to arise but not long enough to allow for retroactive interference to completely deactivate RU associates.

### Model behavior

In the network, the very short 125-ms SOA1 is sufficient to allow for prospective activity elicited by the target-associated Prime 1 in the RU condition (13-ms RU priming; see Table 4). Contrary to the 75-ms SOA2 of Experiment 1, the 250-ms SOA2 of Experiment 2 is sufficient for prospective activity of the associated Prime 2 in the UR condition to allow for a net facilitation of target RTs against proactive interference by the unrelated Prime 1 (12-ms UR priming effects; cf. Table 4). At 125-ms SOA1 and 250-ms SOA2, each of Prime 1 and Prime 2 has time to activate its RU and UR associates, respectively, and has not enough time to generate strong enough proactive and retroactive interference to cancel positive priming by the associated primes in UR and RU conditions, respectively. The simultaneous overall activation of RU and UR associates shown in Experiment 2 and in the current model is in accordance with Lavigne et al.’s (2011; Figures 5b and C2) model simulations.

## Experiment 3: 150 + 100 + 150 + 100

The comparison between Experiments 1 and 2 shows that a partial priming shift arises in the short SOA range. This occurs when primes durations are kept constant and when SOA2 becomes longer than SOA1. The shift was partial in Experiment 2, as shown by the activation of strong and weak RU associates. A possibility for fast retroactive interference on RU priming would rely on longer primes durations for a given short SOA. In the case of proactive interference, the model’s predictions are in accordance with data reporting that processing of a word takes longer when it is presented shortly after another preceding word than when it is presented after a longer delay (Carr, McCauley, Sperber, & Parmelee, 1982). The increased difficulty to process a word after another one could rely on the increased activity of the first word during its processing and shortly after its presentation (e.g., “perceptual response”), as supported by the larger repetition priming reported at short delays (Versace & Nevers, 2003). In multiple-priming protocols, a consequence would be that, for given SOAs, longer durations of the first and second primes would generate strong proactive and retroactive interference due to their perceptual responses. During longer ISIs, the primes are off and generate retrospective activity only and hence also weak retroactive interference. When durations of Primes 1 and 2are identical, we expect retroactive interference to be stronger than proactive interference (see Cowan, 2001; Haarmann & Usher, 2001), leading to UR effects stronger than RU effects. In the experimental literature, cases of equal values of short SOA1 and SOA2 show results varying from significant RU and UR priming (e.g., Angwin, Chenery, Copland, Murdoch, & Silburn, 2005; Balota & Paul, 1996; Chenery, Copland, McGrath, & Savage, 2004) to significant UR priming only (e.g., Masson, 1995; Thérouanne & Denhičre, 2002). In Lavigne et al.’s (2011) Meta-analysis 4, RU priming showed within group heterogeneity inthe range of short SOAs, with an average RU effect size of .17 against .57 when SOA1 was longer than 300 ms. Furthermore, the model also exhibited weak RU priming at short and equal SOAs (see Figure 5B2 of Lavigne et al., 2011), with a variability in the magnitude of RU priming depending on the strength of the association between Prime 1 and the target in the RU condition. The purpose of Experiment 3 was therefore to test the possibility that longer primes durations could support fast retroactive interference when SOA1 and 2 are equal. Given that in Experiment 2 the 250-ms SOA2 allowed for RU priming, Experiment 3 used the same SOA2 but with a Prime 2 duration increased from 50 to 150 ms, to test for the possibility that increased Primes durations could decrease RU priming, generating an early priming shift.

### Participants and protocol

Experiment 3 involved 79 participants and the following protocol:

PD1 = 150 ms, ISI1 = 100 ms (SOA1 = 250 ms), PD2 = 150 ms and ISI2 = 100 ms (SOA2 = 250 ms).

### Results

Data from seven participants who made more than 15% errors were excluded from the analysis. Of the remaining 72 participants, the average error rate was 4.2% and data from error trials were excluded from the analysis. A two-way ANOVA was performed on RTs between the four conditions of Relatedness and the four conditions of Association Strength (Table 4).

Results show a main effect of relatedness, *F_1_*(3, 213) = 5.72, *MSE* = 2338, *p *< .01; an effect of association strength, *F_1_*(3, 213) = 5.56, *MSE* = 2693, *p *< .01; and no significant interaction, *F_1_*(9, 639) = 0.60, *MSE* = 2800, *p* = .80. Planned comparisons indicate significant RR priming effects, *F_1_*(1, 71) = 15.81, *MSE* = 2317.26, *p *< .001, revealing again a re-liable and strong activation of targets associated to both Primes 1 and 2.

Regarding the semantic priming shift, overall RU priming effects are only marginally significant, *F_1_*(1, 71) = 3.41, *MSE* = 1733.14, *p* = .069. Priming by strong RU associates is significant but priming by weak RU associates is not, *F_1_*(1, 71) = 3.97, *MSE* = 1566.49, *p* = .050, and *F_1_*(1, 71) = 0.29, *MSE* = 3090.60, *p* = .59, respectively. UR priming effects are significant, *F_1_*(1, 71) = 8.73, *MSE* = 2058.30, *p *< .01, which is in accordance with retroactive interference being stronger than proactive interference. As a consequence, the processing of Prime 2 activates its associated target (visible in significant priming in the UR condition) and deactivates previously activated associates to Prime 1 (similar to Lavigne et al., 2012, Experiment 2, using 200-ms PD2 and 50-ms ISI2). In Experiment 3, the interaction between RU and UR priming of weak associates is marginally significant, *F_1_*(1, 71) = 3.20, *MSE* = 2342.17, *p* = .078; this corresponds to the activation of Prime 2’s weakly related target in UR conditions but no priming of Prime 1’s weakly related target in the RU condition. This finding is in accordance with the weak and variable RU effects reported in the experimental literature for this range of SOAs. The control and the manipulation of associations strength in the current study allows to identify association strength as a determinant of the variability of RU priming at short SOAs. For the primes durations considered in Experiment 3, the early priming shift occurs on weak associates to the primes, with non-significant priming of RU associates and significant overall priming of UR associates.

### Model behavior

The fact that RU priming arises in Experiment 1 and decreases in Experiment 3 for the same SOA1 is accounted for in the model by a deactivation of RU associates to Prime 1 during the 250-ms SOA2 (due to stronger inhibition of Prime 1 during processing of Prime 2 and to spike frequency adaptation). When comparing Experiments 2 and 3, protocols involved the same 250-ms SOA2, but they differed in two ways: first, SOA1 was longer in Experiment 3, which should have favored RU priming; second, the 150-ms Prime 2 duration was longer in Experiment 3 (50 ms in Experiment 2), which has favored UR priming (15-ms UR priming, see Table 4). The resulting retroactive interference has decreased RU priming (12-ms RU priming, see Table 4). In the network, the longer Prime 2 duration increases the duration of its perceptual response leading to strong inhibitory feedback, which in turn deactivates associates to the first prime in the RU condition. This interference on semantic priming is in accordance with the model’s prediction, showing that retroactive interference generated by the sensory activity during the processing of an item (i.e., here the Prime 2) is stronger than retroactive interference generated by an item but after its offset (Amit et al., 2003; Brunel & Wang, 2001; Haarmann & Usher, 2001). Results of Experiment 3 and the model behavior indicate that the effects of retroactive interference on RU priming are stronger during Prime 2 processing (Experiment 3) than during the ISI2 (Experiment 2). This is in accordance with results on the decrease in interference with the ISI following a word (see e.g., Carr, McCauley, Sperber, & Parmelee, 1982). The present results suggest that the amount of retroactive interference varies not only with SOA2, but also with the respective durations of Prime 2 and ISI2. In addition, and as shown by Experiment 3, the model behavior indicates that the prospective activity of the target-associated Prime 1 in the RU condition highly relies on the strength of the association between Prime 1 and the target (14-ms RU priming by strongly associated Prime 1 and 9-ms RU priming by weakly associated Prime 1; cf. Table 4). As a consequence of the combined effects of prime durations and association strengths, multiple priming effects lead toward a priming shift for weak associates in the range of 250-ms SOAs, with activation of only strong targets associated to Prime1 in the RU condition, and overall activation of the targets associated to Prime 2 in the UR condition.

## Discussion

The current study provides new behavioral data which indicate an early semantic priming shift whose dynamics rely on (a) the respective SOA1 and SOA2, (b) the strength of the association between the target and its related prime, and (c) the durations of the primes.

As it is the case in the range of longer SOAs (Lavigne et al., 2012), UR priming is significant when SOA2 is equal or longer than SOA1 (Experiments 2 and 3). However, Experiment 1 shows for the first time that UR priming, though reported as ubiquitous in a wide range of SOAs in the literature (see Lavigne et al.’s, 2011, meta-analysis), is not fully reliable at the short SOAs considered here and depends on association strength. In single priming protocols involving only one prime where no preceding prime can generate proactive interference, priming effects arise at very short SOAs (Perea & Rosa, 2002; Perea, & Gotor, 1997; Rastle et al., 2000). Experiment 1 indicates that in the same range of short SOAs, multiple-priming protocols allow the proactive interference from the first prime to perturb UR priming. Experiment 2 shows that in the short SOA range, UR priming is significant when SOA2 is longer than SOA1. In this case the UR associates have time to be activated by the prime against proactive interference. Experiment 3 shows for the first time that, even with short SOAs, increased primes durations increase interference that deactivates weak RU associates, generating an early priming shift for weak associates.

The manipulation of the association strength in the current experiments shows that weak associates to the primes are less activated than strong associates, in agreement with experiments on single priming (Abernethy & Coney, 1993; Coney, 2002; Frishkoff, 2007; Hutchinson et al., 2003; for a review, see Chiarello et al., 2003). In addition, the current experiments show that the lower level of activation of weak associates makes them more sensitive to interferences. The cortical network model links the larger priming of strong RU associates in Experiment 3 to a greater prospective activity that better resists to retroactive interference during the processing of the unrelated Prime 2.In addition, the model can account for retroactive interference being stronger than proactive interference because inhibition is stronger during the perceptual response to the second prime than during the retrospective activity of the first prime. As a combined result of the weaker prospective activity of weak associates to the first prime and of stronger retroactive interference on the same weak associates for longer durations of the second prime (Experiment 3), associated targets in the RU condition are less activated than associated targets in UR condition; this corresponds to a semantic priming shift.

The greater sensitivity of weak associates to interference suggests that the early priming shift can concern at least weak associates to the primes and determine the meaning of the sequence of primes. When reading a sequence of words, their meanings activated in working memory are not complete (i.e., only part of their associates are activated) and this changes dynamically as a function of time: (a) activation of associates to the Prime 1 only (cf. Experiment 1), corresponding to a “single meaning” of the first prime only; (b) overall activation of all the associates to the two primes (cf. Experiment 2), that would correspond to “multiple meanings” of the two primes; and (c) activation of only the stronger associates to the Prime 1 (cf. Experiment 3) that would correspond to a “partial meaning” of the prime when the shift is on its way to the activation of all of the associates to the Prime 2 (see also Lavigne et al., 2012). A “single meaning” of the second prime only would rely on a full shift, such as the one that occurs at longer SOA2 (see Lavigne et al., 2012, Experiment 3).

The report of an early shift for weak associates at the behavioral level is in accordance with neurophysiological data on fast inhibitory neurons and rapid retroactive inhibition in cortical networks (e.g., Amit & Brunel, 1997; Brunel & Wang, 2001). The effects of fast inhibition are put in evidence when the Prime 2 duration is long enough to generate retroactive interference on weak associated targets in the RU condition (Experiment 3). Though Prime 2 duration is long enough for inhibition, the 250-ms SOA2 is in the range of short SOAs at which automatic activation but not inhibition is usually considered to take place. Priming and interference are usually reported to depend on qualitatively different though nonexclusive processes of rapid activation and slow inhibition (Mummery, Shallice, & Price, 1999; Neely, 1977, 1991; Neely et al., 1989; Rossell, Bullmore, Williams, & David, 2001; Rossell, Price, & Nobre, 2003). The current results and model show that the early dynamics of the priming shift are subtended by fast retroactive inhibition (see Brunel & Lavigne, 2009). At longer SOAs, the initially fast inhibition continues to build up, slowly, with the increased level and number of associates that become activated (Lavigne et al., 2011, 2012).

The current study suggests that, based on the timing of the processing of the primes, activation based on association strength and unselective feedback inhibition allows the cognitive system to select, at a first time, which RU associates to activate and, at a second time, which UR associates to activate and which RU associates to keep activated against retroactive interference. Results indicate that activations and selections occur early and sequentially at short SOAs after word processing. They arise under the double processing constraints of integration through parallel activation of concepts, and of selection in a working memory with limited capacity. The semantic priming shift can accordingly be considered as an adaptive mechanism that allows the system to update the meanings of the words very rapidly on the basis of their durations (that would contribute to their “perceptive” salience) and of associations strengths (that would contribute to the “semantic relevance” of their associates). Both criteria would then determine the relative levels of activation of RU and UR associates. In the network model, salience and relevance determine the level of perceptual activity of the prime and of prospective activity of its associates, respectively. In turn, salience and relevance related to the processing of a given word in the sequence determine the level of unselective feedback inhibition, which subtends interference on the processing of the other word in the sequence. This corresponds to the fact that the meaning of a word - corresponding to the associates that are activated - is not an all-or-none phenomenon but rather a fast and progressive adaptive phenomenon that depends on the other words in the sequence and on their processing times. Taking these parameters into account allows the cognitive system to adapt the type of meaning activated at a given time to the salience of words processed in the sequence and to the relevance of their associates in memory.

## Appendix A

### Items analyzes for the three experiments

#### Experiment 1

 Results showed a main effect of relatedness, *F_2_*(3, 276) = 1.79,*MSE* = 2643, *p* = .05; a marginally significant effect of association strength, *F_2_*(3, 92) = 2.32, *MSE* = 4680, *p* = .08; and no significant interaction, *F_2_*(9, 276) = 0.48, *MSE* = 2643, *p* = .89. Planned comparisons indicated significant RR priming effects, *F_2_*(1, 92) = 5.18, *MSE* = 1885.36, *p* = .025, showing that during the successive SOA1 and SOA2 the primes or one of them have time to activate the (jointly) associated target. Regarding the semantic priming shift, the overall RU priming effects were significant, *F_2_*(1, 92) = 5.70, *MSE* = 3828, *p* = .019. Priming of strong and of weak associated targets in the RU condition were only marginally significant, *F_2_*(1, 92) = 2.79, *MSE* = 3828.68, *p* = .098, and *F_2_*(1, 92) = 2.91, *MSE* = 3828.68, *p* = .091, respectively. This indicates that the total SOA of 375 ms between Prime 1 and target onset (SOA1 + SOA2) is long enough to allow for overall RU priming despite retroactive interference due to the unrelated Prime 2, in accordance with Lavigne et al.’s (2011) meta-analysis, at least for strongly associated targets in the RU condition, in agreement with the model’s simulations. Regarding the UR condition, the overall priming effect was not significant, *F_2_*(1, 92) = 1.65, *MSE* = 2019.33, *p* = .20, UR priming being marginally significant for strong associates only, *F_2_*(1, 92) = 3.25, *MSE* = 2019.33, *p* = .075.

#### Experiment 2

Results showed a main effect of relatedness, *F_2_*(3, 276) = 3.81, *MSE* = 809, *p* = .011; a marginally significant effect of association strength for participants, *F_2_*(3, 92) = 2.18, *MSE* = 4229, *p* = .096; and no significant interaction, *F_2_*(9, 276) = 0.57, *MSE* = 809, *p* = .83. Planned comparisons indicated significant RR priming effects, *F_2_*(1, 92) = 11.58, *MSE* = 781.125, *p* < .001, showing that, as in Experiment 1, during the successive SOA1 and SOA2 the primes have time to activate their common associated target. Regarding the priming shift, overall RU priming effects were significant, *F_2_*(1, 92) = 4.38, *MSE* = 671.71, *p* = .039. The priming of strongly associated targets in the RU condition was marginally significant but no priming of weakly associated targets in the RU condition was found, *F_2_*(1, 92) = 3.14, *MSE* = 671.71, *p* = .079, and *F_2_*(1, 92) = 1.42, *MSE* = 671.71, *p* = .24, respectively. This indicates that targets associated to Prime 1 can be activated during the short 125-ms SOA1, in accordance with the literature reporting early single priming effects (Perea & Gotor, 1997; Perea & Rosa, 2002; Rastle et al., 2000). With the short 50-ms Prime 2 duration, weakly associated targets in the RU condition continue to be activated against retroactive interference during the 200-ms ISI2. The net UR priming effects were significant in Experiment 2, *F_2_*(1, 92) = 4.94, *MSE* = 720.38, *p* = .029. In Experiment 2 compared to Experiment 1, the priming of weakly associated targets in the UR condition became significant, *F_2_*(1, 92) = 7.37, *MSE* = 720.38, *p* < .01, subtending a priming shift between weakly associated targets, in accordance with the significant interaction between experiments (Experiment 1 vs. Experiment 2) and the type of relation (RU vs. UR) for weak associates, *F_2_*(1, 92) = 5.17, *MSE* = 1483.51, *p* = .025 (the interaction was not significant for strongly associated targets).

#### Experiment 3

 Results showed a main effect of relatedness, *F_2_*(3, 276) = 2.67, *MSE* = 1749, *p* = .048; an effect of association strength, *F_2_*(3, 92) = 3.02, *MSE* = 6699, *p* = .034; and no significant interaction, *F_2_*(9, 276) = 1.19, *MSE* = 1749, *p* = .30. Planned comparisons indicated significant RR priming effects, *F_2_*(1, 92) = 6.98, *MSE* = 1670.57, *p* < .01, revealing again a reliable and strong activation of targets associated to both Primes 1 and 2. Regarding the semantic priming shift, overall RU priming effects were only marginally significant, *F_2_*(1, 92) = 3.21, *MSE* = 2065.24, *p* = .077. Priming of strongly associated targets in the RU condition was significant but priming of weakly associated targets in the RU condition was not, *F_2_*(1, 92) = 5.30, *MSE* = 2065.24, *p* = .024, and *F_2_*(1, 92) = 0.053, *MSE* = 2065.24, *p* = .82, respectively. UR priming effects were significant, *F_2_*(1, 92) = 7.27, *MSE* = 1189.76, *p* < .01, which is in accordance with retroactive interference being stronger than proactive interference.
